# HOW STATES CHANGED REGULATORY REQUIREMENTS FOR ASSISTED LIVING STAFF SINCE 2003

**DOI:** 10.1093/geroni/igz038.1655

**Published:** 2019-11-08

**Authors:** Paula Carder, Sarah Dys

**Affiliations:** 1 Portland State University Institute on Aging, Portland, Oregon, United States; 2 Portland State University, Portland, Oregon, United States

## Abstract

In 2003, the Assisted Living Workgroup (ALW) published quality improvement recommendations for states’ regulations, including 26 regarding staffing/workforce. We reviewed states’ 2003 and current regulations to identify the presence of ALW standards. Over half of states’ regulations reflect 7 of the 26 staffing/workforce recommendations. Those most often added after 2003 concern criminal background checks, with a 58.8 percent increase in states that added federal background checks and use of criminal background checks to inform hiring. At least 40 states’ regulations reflect the ALW recommendations for administrator and direct care staff training. Very few states require staff performance evaluations (n=13), human resource policies to improve retention (n=1), or management practices to improve retention (0). The 10 ALW recommendations concerning staff who administer medications have been adopted by fewer than 23 states. These findings can inform future policy analysis and research on staffing/workforce in assisted living communities.

